# Quantitative Anatomical Studies

**DOI:** 10.1155/2015/781590

**Published:** 2015-08-31

**Authors:** Ilker Ercan, Levent Sarikcioglu, Heather F. Smith, Juan A. Sanchis-Gimeno, Tuncay Peker, Gulsum Ozyigit

**Affiliations:** ^1^Department of Biostatistics, Medical Faculty, Uludag University, 16059 Bursa, Turkey; ^2^Department of Anatomy, Medical Faculty, Akdeniz University, Antalya, Turkey; ^3^Department of Anatomy, Midwestern University, Glendale, AZ 85308, USA; ^4^Department of Anatomy and Human Embryology, University of Valencia, Valencia, Spain; ^5^Department of Anatomy, Medical Faculty, Gazi University, Ankara, Turkey; ^6^Department of Anatomy, Faculty of Veterinary Sciences, Uludag University, Bursa, Turkey

This special issue of this journal highlights new developments mainly in the field of quantification of the data of anatomical studies. In this special issue, we reviewed and edited seventeen articles from broad ranges of anatomical studies.

N. Utkualp and I. Ercan reviewed anthropometric measurement usage in medical sciences from Ancient Egyptian, Greek, and Roman civilizations to those from modern medicine. They also stressed contributions of the well-known scientists to recent diagnostic methods.

S. Liao et al. studied the fundamental problem of automatically segmenting teeth in dental mesh models into individual tooth objects and they built a novel dental-targeted harmonic field, which is able to automatically segment all teeth only once under a uniform harmonic field computation. They stressed that extensive experiments and quantitative analysis demonstrated the effectiveness of the method in terms of accuracy, robustness, and efficiency. G. Lo Giudice et al. performed a quantitative evaluation of dentin morphology in the root canal surface of premolars in paediatric-aged patients and showed that dentinal structure varied in the different root canal portions.

L. Tomak et al. developed a chart for monitoring the inclination of the acetabular component after total hip replacement surgery. They demonstrated that time-weighted quality control charts can also be used in the field of medicine and that they allow for a faster visual decision. N. Hauser et al. determined incidence and position of the fabella in a central European population and assessed how to better estimate clinical appearances. They also described the biomechanical impact of the small sesamoid bone in its interaction with the femur, in order to determine a possible pressure distribution. O. Louis et al. compared MRI-derived bone mineral density (BMD) measured by advanced Bone Analysis Applications software and biomechanical tests. They found that MRI-derived BMD correlates with failure load to an extent, comparable to BMD estimates derived from classical bone densitometry techniques (pQCT or DXA).

H.-J. Cho et al. compared morphometric characteristics of Korean femora by geometric computation and calculated the size of the medullary canal for implant stem and intramedullary device design. They provided references for physical and forensic anthropology of a Korean population. P. W. L. ten Berg et al. studied three-dimensional assessment of bilateral symmetry of healthy bilateral scaphoid pairs in terms of three translational and three rotational parameters. They suggested that the contralateral scaphoid can serve as a reference in corrective surgery. S. Kim et al. investigated muscle architecture throughout the volume of the supraspinatus muscle by using three-dimensional model of fiber bundle architecture within the anterior and posterior regions of pathologic supraspinatus muscle. They found distinct patterns of change and suggested that their model can be incorporated with existing shoulder models to be used for biomechanical analysis in different patient populations with supraspinatus tendon pathologies. B. Felix-Patrício compared point-counting method and color-based segmentation method in surface density data of penile corpus cavernosum trabecular smooth muscle and gave some cues in final interpretation of the results.

A. Sezer et al. performed multiple comparisons of age groups in bone mineral density under heteroscedasticity and reported their results in non-Hispanic whites, non-Hispanic blacks, and Mexican Americans. D. Dalal and H. F. Smith studied developmental changes in morphology of the middle and posterior external cranial bases in modern* Homo sapiens* and reported that the basicranium, occipital, and temporal regions reflected genetic distances among populations in childhood and adolescence.

M. Szpinda et al. assessed liver volumes in human fetuses of both sexes by using anatomical, hydrostatic, and statistical methods and proposed a formulation to calculate liver volume for evaluation of normal hepatic growth and early diagnosis of fetal microsomia and macrosomia. In another study, M. Szpinda et al. studied growing lungs in human fetuses and described relationships of lung dimensions in normative pulmonary growth and the diagnosis of pulmonary hypoplasia.

R. A. Aversi-Ferreira et al. reanalyzed previously published data in comparative anatomy statistics and suggested some cues for accurate analysis of anatomical data. J. W. Kim et al. reported their preliminary observations on sensitivity and specificity of magnetization transfer asymmetry for imaging myelin of the rat brain at high field. They found that magnetization transfer asymmetry can be a good biomarker for imaging myelination with better specificity than and similar sensitivity to magnetization transfer ratio. B. Ozdemir et al. compared the association of aortic diameters with coronary artery disease severity and albumin excretion. They found that patients with coronary artery disease had higher systolic blood pressure, pulse pressure, aortic systolic and diastolic pressure, and albumin excretion rate and had lower aortic distensibility.

We tried to summarize key points of the articles of this special issue. Readers of the special issue will have a chance to review a broad spectrum of quantification efforts of anatomical data and advances in anatomical quantification techniques. Accurate analysis in any science is a fundamental topic in study design, data collection, and drawing of interpretations from data.

As always, we wish you good reading.

